# Supplementary material for: Prediction of Coronary Artery Calcium Score Using Machine Learning in a Healthy Population
Source: J Pers Med. 2020 Aug 20;10(3):96. doi: 10.3390/jpm10030096 (PMC7565334; doi:10.3390/jpm10030096)
Supplement: Supplementary file 1 [file jpm-10-00096-s001.pdf]

Supplemental Table 1. The comparison of input features between training and test dataset.

	Training (n = 1,494)	Test (n = 639)	p value
Age, years	55.4±11.2	55.2±11.5	0.651
Male	1,027 (68.7)	456 (71.4)	0.249
Height, cm	166.0±8.7	166.5±9.1	0.228
Weight, Kg	69.0±11.8	69.3 ± 11.9	0.637
Abdominal circumference, cm	85.5±9.3	85.2 ± 9.0	0.554
BMI, Kg/m^2	25.0±3.1	24.9 ± 2.9	0.576
BPsystolic, mmHg	121.4±14.8	122.2 ± 14.3	0.214
BPdiastolic, mmHg	73.3±11.1	73.8 ± 11.3	0.292
hsCRP, IU/L	1.3±2.5	1.2 ± 2.0	0.418
FBS, mg/dL	103.4±25.8	102.3 ± 22.9	0.331
A1c, %	5.7±0.8	5.6 ± 0.8	0.500
Bilirubin (total), mg/dL	0.9±0.3	0.9 ± 0.3	0.845
Bilirubin (direct), mg/dL	0.2±0.1	0.2 ± 0.1	0.223
gamma-GT, IU/L	45.3±62.3	43.8 ± 54.6	0.603
ALP, IU/L	72.2±24.6	73.0±26.6	0.563
LDH, IU/L	217.7±82.2	221.9±85.5	0.284
AST, IU/L	29.2±32.3	28.2±16.1	0.344
ALT, IU/L	31.0±46.6	30.7±24.6	0.863
BUN, mg/dL	13.6±3.4	13.6±3.2	0.613
Creatinine, mg/dL	0.9±0.3	0.9±0.2	0.154
eGFR, mL/min	84.5±27.3	85.9±26.9	0.301
Total cholesterol, mg/dL	193.7±38.2	195.4±40.2	0.370
TG, mg/dL	147.0±104.4	137.0±84.6	0.020
HDL, mg/dL	52.5±13.1	52.3±12.9	0.796
LDL, mg/dL	107.7±51.6	107.4±55.3	0.883
WBC, 10^3/μL	5.7±1.5	5.7±1.6	0.428
Hemoglobin, g/dL	14.7±1.4	14.8±1.5	0.189
MCV, fL	91.7±4.3	91.7±4.4	0.842
Platelet count, 10^3/μL	241.7±48.9	243.4±49.3	0.455

Values were presented as mean ± standard deviation or number (column percent) as appropriate.

CACS, coronary artery calcium score; BMI, body mass index, BP, blood pressure; hsCRP, high sensitivity C-reactive protein; FBS, fasting blood sugar; A1c, glycated hemoglobin; gamma-GT, gamma-glutamyl transferase; ALP, alkaline phosphatase; LDH, Lactate dehydrogenase; AST, Aspartate transaminase; ALT, alanine aminotransferase; BUN, blood urea nitrogen; eGFR, estimated glomerular filtration rate; TG, triglycerides; HDL, high-density lipoprotein; LDL, low-density lipoprotein; WBC, white blood cell; MCV, mean corpuscular volume; IU, international unit.

Supplemental Table 2. Results of the binary logistic regression analysis of the training dataset.

	OR (95%CI)	p value
Age, years	1.13 (1.10–1.16)	<0.001
Male	3.31 (1.56–7.03)	0.002
Height, cm	0.91 (0.76–1.08)	0.282
Weight, Kg	1.12 (0.92–1.38)	0.265
Abdominal circumference, cm	1.03 (0.99–1.07)	0.158
BMI, Kg/m^2	0.70 (0.40–1.22)	0.209
BPsystolic, mmHg	1.01 (0.99–1.03)	0.162
BPdiastolic, mmHg	1.01 (0.99–1.04)	0.309
hsCRP, IU/L	0.91 (0.80–1.05)	0.202
FBS, mg/dL	1.00 (0.99–1.01)	0.531
A1c, %	1.23 (0.90–1.68)	0.194
Bilirubin (total), mg/dL	1.02 (0.35–2.93)	0.975
Bilirubin (direct), mg/dL	0.47 (0.02–12.89)	0.658
gamma-GT, IU/L	1.00 (1.00–1.01)	0.193
ALP, IU/L	1.00 (0.99–1.01)	0.869
LDH, IU/L	1.00 (1.00–1.00)	0.538
AST, IU/L	1.01 (1.00–1.03)	0.181
ALT, IU/L	0.98 (1.00–1.13)	0.063
BUN, mg/dL	1.06 (1.00–1.13)	0.054
Creatinine, mg/dL	0.99 (0.47–2.12)	0.988
eGFR, mL/min	1.01 (1.00–1.02)	0.216
Total cholesterol, mg/dL	1.00 (0.99–1.01)	0.729
TG, mg/dL	1.00 (1.00–1.00)	0.624
HDL, mg/dL	0.99 (0.98–1.01)	0.376
LDL, mg/dL	1.00 (0.99–1.00)	0.257
WBC, $10^3/\mu L$	1.01 (0.89–1.14)	0.891
Hemoglobin, g/dL	0.88 (0.73–1.06)	0.181
MCV, fL	1.00 (0.96–1.05)	0.912
Platelet count, $10^3/\mu L$	1.00 (1.00–1.00)	0.883

OR, odds ratio; CI, confidence interval; BMI, body mass index, BP, blood pressure; hsCRP, high sensitivity C-reactive protein; FBS, fasting blood sugar; A1c, glycated hemoglobin; gamma-GT, gamma-glutamyl transferase; ALP, alkaline phosphatase; LDH, Lactate dehydrogenase; AST, Aspartate transaminase; ALT, alanine aminotransferase; BUN, blood urea nitrogen; eGFR, estimated glomerular filtration rate; TG, triglycerides; HDL, high-density lipoprotein; LDL, low-density lipoprotein; WBC, white blood cell; MCV, mean corpuscular volume; IU, international unit.

Supplemental Table 3. Parameter optimization of each machine learning algorithms.

	BLR	catboost	xgboost
5-fold cross-validation	yes	yes	yes
Grid search	no	yes	yes
Scaling/normalization	no	no	no
Maximal depth	-	8	12
Kappa	-	0.052890996	-
Learning rate	-	0.1	-
Loss	-	rsm (0.95)	logloss
Border count	-	64	-
iteration	-	10	50
Objectives	-	L2 regularization (0.1)	binary:logistic
eta	-	-	0.1
gamma	-	-	0.1

BLR, binary logistic regression; rsm, random subspace method.
